# Virtual Care for Indigenous Populations in Canada, the United States, Australia, and New Zealand: Protocol for a Scoping Review

**DOI:** 10.2196/21860

**Published:** 2020-12-01

**Authors:** Pat Camp, Mirha Girt, Alix Wells, Adeeb Malas, Maryke Peter, Stephanie Crosbie, Travis Holyk

**Affiliations:** 1 Centre for Heart Lung Innovation University of British Columbia Vancouver, BC Canada; 2 Department of Physical Therapy University of British Columbia Vancouver, BC Canada; 3 Carrier Sekani Family Services University of British Columbia Prince George, BC Canada; 4 Faculty of Medicine University of British Columbia Vancouver, BC Canada; 5 No affiliation Vancouver, BC Canada

**Keywords:** virtual care, Indigenous, Indigenous health, accessibility, cultural competency, feasibility, acceptability, utility

## Abstract

**Background:**

Indigenous people in Canada, the United States, Australia, and New Zealand experience an increased burden of chronic diseases compared to non-Indigenous people in these countries. Lack of necessary services and culturally relevant care for Indigenous people contributes to this burden. Many Indigenous communities have implemented systems, such as virtual care, to improve chronic disease management. Virtual care has extended beyond videoconferencing to include more advanced technologies, such as remote biometric monitoring devices. However, given the historical and ongoing Western intrusion into Indigenous day to day life, these technologies may seem more invasive and thus require additional research on their acceptability and utility within Indigenous populations.

**Objective:**

The objective of this paper is to present the protocol for a scoping review, which aims to map existing evidence. This study is based on the following guiding research question: What are the characteristics of virtual care use by Indigenous adult populations in Canada, the United States, Australia, and New Zealand? The subquestions are related to the technology used, health conditions and nature of the virtual care, cultural safety, and key concepts for effective use.

**Methods:**

This scoping review protocol is informed by the methodology described by the Joanna Briggs Institute and is supplemented by the frameworks proposed by Arksey and O’Malley and Levac et al. A search for published and gray literature, written in English, and published between 2000 and present will be completed utilizing electronic databases and search engines, including MEDLINE, CINAHL, Embase, Indigenous Peoples of North America, Australian Indigenous HealthInfoNet, Informit, and Native Health Database. Search results will be uploaded to the review software, Covidence, for title and abstract screening before full-text screening begins. This process will be repeated for gray literature. Upon completion, a data abstraction tool will organize the relevant information into categorical formations.

**Results:**

The search strategy has been confirmed, and the screening of titles and abstracts is underway. As of October 2020, we have identified over 300 articles for full-text screening.

**Conclusions:**

Previous reviews have addressed virtual care within Indigenous communities. However, new virtual care technologies have since emerged; subsequently, additional literature has been published. Mapping and synthesizing this literature will inform new directions for research and discussion.

**International Registered Report Identifier (IRRID):**

PRR1-10.2196/21860

## Introduction

Indigenous people in Canada, the United States, Australia, and New Zealand carry an increased disease burden of many chronic illnesses compared to non-Indigenous residents [1]. The reasons for this are multifactorial. The common history of colonialism, residential school systems, and forced removal of Indigenous children from their families resulted in an intergenerational trauma for many people [2,3]. In addition, the ongoing discrimination, lack of appropriate services, and limited culturally relevant care, especially for Indigenous people in remote and rural locations, contribute to worse health outcomes for Indigenous people in these countries [2-4].

To address these and other harms, federal governments in these countries are working with Indigenous nations and groups to develop policies to improve health. The report of the Truth and Reconciliation Commission of Canada [2] announced several calls to action to improve the health of First Nations people in Canada, while simultaneously acknowledging the Indigenous rights in determining how research and health care will be conducted, including “a focus on chronic diseases and the availability of appropriate health services.” The *Truth Telling Symposium Report* by Reconciliation Australia and the Healing Foundation [3] acknowledged past and current harms and called for truth telling to improve relationships and shape policy. In New Zealand, the Waitangi Tribunal [4] provides a mechanism where treaty violations, including those related to health and welfare, are addressed. The United States signed into law the Apology to Native Peoples of the United States [5]. Within many of these documents, there is an acknowledgement of the need for culturally safe health care initiatives to address health care inequities related to acute and chronic disease health care.

Many Indigenous communities have already implemented innovative systems, such as virtual care services, to improve chronic disease management for their members. For example, Carrier Sekani Family Services (CSFS) is a nonprofit, First Nations health care provider in Canada with the mandate to provide health, social, and research services on behalf of its member First Nations. CSFS recognized that their member communities were disadvantaged when trying to access timely and culturally sensitive health care for acute and chronic disease management. In 2010, CSFS developed a videoconferencing telehealth system that connects patients and providers so that communities have regular access to a primary care physician most days of the week. An evaluation of the CSFS system [6] found that 52% of survey respondents had used the service at least once, and of those respondents, 83% attended more doctor’s appointments, and 78% had fewer out-of-community trips for health care, compared to before the service was introduced. Other jurisdictions have similar programs and have reported similar benefits [7-11].

Virtual care, however, is more than videoconferencing. Additional technologies such as internet-delivered care, remote biometric monitoring, wearables, and smartphone apps are now used in virtual care. These technologies have the potential to support the ongoing management and transitions in care of people with chronic diseases living in remote and rural communities. Additional research into the acceptability and utility of virtual care technologies for Indigenous people is necessary, given the past and current harms of Western intrusion (colonization, creation of reserve lands and surveillance, and residential school systems) into the Indigenous day to day life. Monitoring devices may seem all the more invasive to Indigenous peoples, and there may be concerns about the privacy of data, ease of using the system, reliability of devices, suitability of technology where remoteness impacts connectivity, and concerns about technology replacing genuine relationships between patients and care providers. The potential benefits of increasing technology in virtual care must be weighed against individual or community harms that may result from increased surveillance.

Indigenous communities, researchers, and advocates have explored how virtual care technology, from basic to more advanced, can be used to improve health outcomes of Indigenous peoples as well as to identify the limitations of these systems, issues related to cultural safety, and current gaps in information. Their knowledge has been published in peer-reviewed journals, community reports, best practice guidelines, and government documents and includes insights into barriers, facilitators, and key principles for telehealth for Indigenous peoples. We undertook an environmental scan of the published and gray literature to confirm the feasibility of a scoping review and to test keywords and search strategies. A preliminary search of 3 databases using relevant search terms, plus a gray literature search, revealed at least 50 documents related to virtual care in Indigenous communities. A brief review of the papers identified relevant topics, including the benefits of telehealth (eg, reduces alienation), the challenges (eg, requires reliable internet services and sustainable infrastructure support), and important principles (eg, requires culturally appropriate care and transparency of the data). A structured scoping review of this knowledge would provide a mapping of the current knowledge, identification of key concepts, description of how virtual care (including invasive technologies) is used, and recommendations for future research and care. The purpose of this paper is to describe the protocol for the scoping review of virtual care for Indigenous populations in Canada, the United States, Australia, and New Zealand.

## Methods

This scoping review protocol is informed by the methodology devised by the Joanna Briggs Institute [12]. This methodology is supplemented by the frameworks proposed by Arksey and O’Malley [13] and Levac et al [14].

### Consultation

Consultation is an important part of the scoping review process. The executive director of the CSFS primary care services (TH) is an investigator on this project and participated in the development of the search strategy. In addition, we will convene meetings throughout the review process with the entire CSFS virtual care team to gain feedback on the document summaries, interpretation of the results, and creation of the knowledge translation materials.

### Identifying the Research Questions

Using the Population-Concept-Context (PCC) framework, the review will focus on Indigenous adults’ (Population) utilization of virtual care (Concept) in Canada, the United States, Australia, and New Zealand (Context). These geographical areas are relevant due to many similarities of these locations in terms of their history of European colonization, their worldviews, and the leadership of Indigenous peoples in virtual care. We identified 13 subquestions as detailed in Table 1. We considered virtual care and its equivalents as health care whereby health care providers interact with their patients through technology, including video, audio, messaging, the internet, apps, or wearables.

**Table 1:** Guiding research question and subquestions.

Category of questions		Questions
Guiding research question		What are the characteristics of virtual care use by Indigenous adult populations in Canada, the United States, Australia, and New Zealand?
**Subquestions**		
	General	What is the total number of documents published each year? What terms or keywords are being used to describe these documents? What are the characteristics of the authors and institutions, communities, or agencies producing the knowledge? Where are the authors located, according to their affiliations? What is the geographical scope of the knowledge?
	Technology	What types of virtual care technology are being used? What is the chronological evolution of virtual care?
	Health condition and virtual care intervention	For what health conditions is virtual care being used? What are the key elements of the virtual care provided? Who provides the intervention? (remote, local, academic, or community) What is the involvement of Indigenous people in the provision of virtual care—as a provider, organization, developer?
	Cultural safety	What are the key concepts of cultural safety when using virtual care?
	Effective use	What are the key concepts of the effective use of virtual care?

### Identifying and Selecting Relevant Studies and Documents

#### Published Literature: Search Strategy

The following licensed electronic databases (from 2000 to present) will be used to systematically look for published literature: (1) MEDLINE, (2) EMBASE, (3) CINAHL, (4) PubMed, (5) Indigenous Peoples of North America, (6) Indigenous Studies Portal, (7) Informit, and (8) Native Health Database. The sources selected will be limited to papers in English. The search strategy, based on the PCC framework, focuses on Indigenous peoples receiving virtual care in Canada, the United States, Australia, and New Zealand. Table 2 lists the general search strategy; this strategy will be adapted to each database as appropriate.

**Table 2:** General search strategy.

Elements of the PCC^a^ framework and line		Search terms
**Population**		
	1	exp Indians, North American/
	2	exp Alaska Natives/
	3	exp Inuits/
	4	exp Indigenous Peoples/
	5	exp Health Services, Indigenous/
	6	exp Oceanic Ancestry Group/
	7	(Indigenous adj3 Australia*) OR (aborigin* adj3 Australia*) OR (Torres Strait Islander*) OR (Maori).mp
	8	(Indigenous OR First Nation* OR Inuit* OR Metis OR Aborigin* OR (Native* adj3 America*) OR American Indian* OR America* adj3 Native* or Amerind* or (Alaska* adj3 Native*)).mp
	9	1 OR 2 OR 3 OR 4 OR 5 OR 6 OR 7 OR 8
**Concept**		
	10	exp Telemedicine/
	11	exp Telemetry/
	12	exp Telenursing/
	13	exp Telerehabilitation/
	14	exp Mobile Applications/
	15	exp Smartphone/
	16	exp Cell Phone/
	17	exp Biometry/
	18	exp Biometric Identification/
	19	exp Wearable Electronic Device/
	20	exp Biosensing Techniques/
	21	exp Self-Help Devices/
	22	exp Monitoring, Physiologic/
	23	(telehealth* OR tele-health* OR telemedicine OR tele-medicine OR tele-psychiatry OR teleophthalmology OR telecare OR tele-care OR telenurs* OR tele-nurs* OR mobile health* OR ehealth OR e-health OR mhealth OR m-health OR telemonitor* OR tele-monitor* OR telerehabilitat* OR tele-rehabilitat* OR remote medicine OR remote health* OR distance medicine OR digital health* OR remote biometr* OR (remote adj2 monitor*) OR (virtual adj2 care) OR wearable* OR smart device* OR health sensor* OR health monitor* OR biosensor* OR biometric* OR mobile technolog* OR mobile monitor* OR smartphone* OR smart phone* OR cellphone* OR cell phone* OR mobile phone* OR app OR apps OR Fitbit* OR fitness tracker*).mp
	24	10 OR 11 OR 12 OR 13 OR 14 OR 15 OR 16 OR 17 OR 18 OR 19 OR 20 OR 21 OR 22 OR 23
	25	9 AND 24
Context		Canada, the United States, Australia, New Zealand (applied at the full-text review stage)

^a^PCC: Population-Concept-Context.

#### Gray Literature: Search Strategy

A gray literature search (from 2000 to present), using the search strategy in Table 2, will be conducted in the institutional and governmental electronic databases and search tools, including (1) The New York Academy of Medicine, (2) OpenGrey, (3) Canadian Agency for Drugs and Technologies in Health, (4) Canadian Institute for Health Information, (5) Health Canada, Government of Canada, (6) OAIster, (7) Agency for Healthcare Research and Quality, (8) Circumpolar Health, (9) Department of Health, Australia, and (10) Australian Indigenous HealthInfoNet. An additional search of Google and Google Scholar will be conducted with the first 50 relevancy-ranked results reviewed. The resources selected will be limited to those in English.

Selected documents or studies will have a specific focus on the use of virtual care in Indigenous populations in Canada, the United States, Australia, and New Zealand and will have been published or made available from 2000 to present. The studies will not be limited by the study design, age group, or type of health condition addressed via virtual care. For gray literature, the document types selected for inclusion in this review will be limited to conference proceedings, government or agency reports, practice guidelines, annual reports, program evaluations, literature reviews, and policy papers.

All citations will be uploaded to the review software Covidence. Each title and abstract will be screened by 2 independent team members who will make a yes or no selection. A third team member will resolve any discrepancies. We will then obtain the full-text articles for all selected citations. Each full-text article will be independently reviewed by 2 team members with discrepancies resolved by a third team member.

For the gray literature, we will upload documents to Covidence and will use the same procedure for title screening as described for the published literature. For those search results that cannot be uploaded to Covidence, a document will be generated that lists each result. These will all be independently reviewed by 2 team members with discrepancies resolved by a third team member. For the Google search, search results may change from one minute to the next. Therefore, a team member will generate the search results and will review the first 50 sources. This same search result list will then be reviewed separately by a second team member, and discrepancies will be resolved by a third team member.

### Charting and Synthesizing the Data

A document to abstract the data (Textbox 1) from selected documents will be designed, pilot tested, and used by 2 team members to independently extract data from the selected documents. If there is any disagreement between the 2 team members, it will be resolved through the help of a third team member.

**Data abstraction for the guiding research question and subquestions.**

Summary

- Title
- Authors
- Author’s affiliation
- Type of literature
- Keywords
- Study Location
- Participants in study
- Study population
- Objective of study

Subquestion 1: technology and intervention

- Technology being used
- Intervention—description, provider, agencies involved
- Involvement of Indigenous peoples

Subquestion 2: health condition

- Type of health condition

Subquestion 3: cultural safety

- Key concepts discussed

Subquestion 4: effective use/outcomes described

- Key concepts discussed

The abstracted information will be summarized by subquestions using tables, bar graphs, and narratives where appropriate.

## Results

The search strategy has been confirmed, and we are reviewing the published and gray literature. As of October 2020, we have identified 2928 titles and abstracts from MEDLINE, EMBASE, and CINAHL; 395 of those have moved to the stage of full-text screening. We are completing the gray literature database searches and to date have identified 2645 gray literature documents for screening.

## Discussion

Previous work has reviewed virtual care in Indigenous communities. In their 2010 report for the British Columbia Alliance on Telehealth Policy and Research, Lavoie and colleagues [15] reviewed available published and gray literature pertinent to telehealth development in First Nations communities within British Columbia, Canada. Findings from the review demonstrated the importance of the development and implementation of contextualized telehealth delivery in First Nations communities. Others have reviewed telemedicine in Indigenous populations, but those studies are often focused on one country or only included knowledge from peer-reviewed journals [16,17]. A reflective analysis of this wide breadth of knowledge across several relevant topics will synthesize the knowledge from many knowledge sources from Canada, the United States, Australia, and New Zealand and will reveal new directions for research and discussion.

## Abbreviations

CSFS: Carrier Sekani Family Services
PCC: Population-Concept-Context
